# Incorporación de equidad en salud en la investigación sobre implementación: revisión de los modelos conceptuales

**DOI:** 10.26633/RPSP.2017.126

**Published:** 2017-12-05

**Authors:** Javier Eslava-Schmalbach, Nathaly Garzón-Orjuela, Vanessa Elias, Ludovic Reveiz

**Affiliations:** 1 Hospital Universitario Nacional de Colombia Grupo de Equidad en Salud, Facultad de Medicina, Universidad Nacional de Colombia Bogotá Colombia Hospital Universitario Nacional de Colombia, Grupo de Equidad en Salud, Facultad de Medicina, Universidad Nacional de Colombia, Bogotá, Colombia.; 2 Organización Panamericana de la Salud Organización Panamericana de la Salud Washington, D.C. Estados Unidos de América Organización Panamericana de la Salud, Washington, D.C., Estados Unidos de América.

**Keywords:** Modelos teóricos, investigación en servicios de salud, equidad en salud, Models, theoretical, health services research, health equity, Modelos teóricos, pesquisa sobre serviços de saúde, equidade em saúde

## Abstract

**Objetivo.:**

Buscar y elaborar una síntesis sistemática de los marcos o modelos conceptuales que incorporan aspectos de equidad en salud en implementación de la investigación.

**Métodos.:**

Búsqueda sistemática en Medline-Pubmed, Embase y Lilacs (1965-2016) y Scopus (1998-2016). Además, se utilizó una estrategia en bola de nieve y búsqueda de la literatura gris. Se evaluó el tipo de artículo y los elementos que se incluyeron sobre investigación de la implementación (IR, por sus siglas en inglés), la ciencia de la implementación y equidad en salud.

**Resultados.:**

Se encontraron 701 artículos, de los cuales 100 se incluyeron para revisión de relevancia. De estos, quedaron 19 artículos relacionados con marcos conceptuales: 12 fueron generales, cinco sobre disparidades étnicas o raciales y dos relacionados con salud infantil. Las categorías más frecuentes fueron: financiación, infraestructura, abogacía, calidad, barreras internas y cobertura. Las menos frecuentes fueron: otros sectores, las necesidades de los pacientes externos, el estado de salud y la evaluación del impacto sobre la equidad.

**Conclusiones.:**

Para disminuir las brechas en salud y con ellas las inequidades en salud, se hace necesario contar con un marco consolidado de IR en el que se incluyan los aspectos de equidad en salud. Este marco facilitaría mejorar los procesos de implementación de las intervenciones, los servicios y los programas de salud.

## Abstract (en)

**Objective.:**

Search for conceptual frameworks or models that incorporate aspects of health equity into implementation research and prepare a systematic synthesis of them.

**Methods.:**

A systematic search was done of MEDLINE-PubMed, Embase, and LILACS (1965–2016), and Scopus (1998–2016) databases, employing, in addition, a snowball strategy and gray literature search. Article type was assessed, along with elements that were included on implementation research, the science of implementation, and health equity.

**Results.:**

A total of 701 articles were found, of which 100 were included for review of relevance. Of these, 19 articles related to conceptual frameworks: 12 were general, five were on ethnic or racial disparities, and two related to child health. The most frequent categories were financing, infrastructure, advocacy, quality, internal barriers, and coverage. The least frequent were other sectors, outpatient needs, health status, and equity impact assessment.

**Conclusions.:**

In order to reduce health gaps and with them health inequities, it becomes necessary to have a consolidated implementation research framework that includes aspects of health equity. This framework would facilitate improving implementation processes for interventions, services, and health programs.

## RESUMO

**Objetivo.:**

Fazer uma busca e revisão sistemática dos marcos ou modelos conceituais que abrangem aspectos de igualdade em saúde em pesquisa de implementação.

**Métodos.:**

Busca sistemática nas bases de dados MEDLINE-PubMed, Embase e LILACS (1965–2016) e Scopus (1998–2016) com o uso de estratégia de amostragem em bola de neve e busca da literatura cinzenta. Foram avaliados o tipo de artigo e os elementos contidos sobre pesquisa de implementação (implementation research, IR), ciência de implementação e igualdade em saúde.

**Resultados.:**

Foram identificados 701 artigos, dos quais 100 foram incluídos na revisão. Destes, 19 artigos estavam relacionados aos marcos conceituais: 12 gerais, cinco sobre disparidades étnicas ou raciais e dois relacionados com saúde infantil. As categorias mais frequentes dos marcos conceituais foram: financiamento, infraestrutura, defesa da causa, qualidade, barreiras internas e cobertura. As menos frequentes foram: outros setores, necessidades dos pacientes ambulatoriais, estado de saúde e avaliação do impacto na equidade.

**Conclusões.:**

Para reduzir as lacunas em saúde e as desigualdades em saúde, faz-se necessário dispor de um modelo consolidado de pesquisa de implementação no qual sejam incluídos os aspectos de igualdade em saúde. Este modelo contribuiria para melhorar os processos de implementação das intervenções, serviços e programas de saúde.

La investigación de la implementación (IR, por sus siglas en inglés por *implementation research*) se describe como el estudio científico de los procesos de implementación de intervenciones, servicios o programas de salud, en los cuales se incluyen los factores contextuales que afectan o podrían afectar dichos procesos de implementación (1).

La IR hace parte de la investigación en servicios y políticas en salud (*health policy and system research*) y se enfoca en la ciencia de la implementación (*implementation science*), la cual busca mejorar el desempeño de los sistemas de salud mediante el estudio sistemático de las estrategias, procesos de implementación y factores contextuales, a partir de intervenciones, servicios o programas que tienen evidencia de efectividad (2).

El fundamento de la IR es la existencia de brechas entre lo que se llama ciencia y la provisión del servicio (*science to service* *gap*) o del conocimiento a la acción (*know* *to do gap* o *knowledge to action gap*) o brecha en la provisión del servicio (*delivery service gap*) (3, 4).

En 2015, Davidson et al. publicaron una revisión sistemática relacionada con los modelos de conocimiento a la acción e implicaciones para promover la equidad en salud. Se identificaron y evaluaron 48 modelos de conocimiento a la acción basados en seis características de equidad en salud que pueden tener utilidad para apoyar la equidad en salud. Los conocimientos previos a los modelos o marcos de acción pueden ayudar a orientar la trasferencia de conocimientos para apoyar la acción sobre los determinantes sociales de la salud y la equidad en salud (5).

Para facilitar el proceso sistemático de la IR, se ha propuesto un marco consolidado que incorpora cinco elementos: características de la intervención, el medio externo, el medio interno, las características de los individuos involucrados y el proceso de implementación (1).

Para el desarrollo de este artículo se acogió el concepto de inequidad en salud definida por Whitehead, como la presencia de aquellas desigualdades en salud que son innecesarias, evitables y, además, injustas (6). Aunque esta definición es ampliamente utilizada, la documentación sobre su operativización en la implementación de intervenciones, guías de prática clínica o programas de salud es bastante escasa. La Organización Panamericana de la Salud (OPS), por ejemplo, propone de manera teórica un abordaje intersectorial de la salud, bajo las perspectivas del mejoramiento de la salud de los más vulnerables, de la disminución de las brechas existentes, o del abordaje directo del gradiente en salud dado por la posición socioeconómica (7). Sin embargo, la operativización de esta propuesta no es clara al momento de abordar la implementación.

Por otro lado, en el marco consolidado para IR, si bien se incluyen las características de los individuos involucrados y el medio externo (1, 7), no se mencionan de manera explícita las consideraciones de equidad o inequidad en salud, o cómo las intervenciones o programas podrían afectar de manera positiva o negativa las desigualdades evitables e injustas en salud. Aunque la ciencia de la implementación se enfoca en los desenlaces de implementación relacionados con el proceso mismo de la implementación y sus factores contextuales, los efectos inmediatos o tardíos sobre los desenlaces en salud, o los aspectos de la intervención misma que afecten la equidad en salud no están explícitamente considerados dentro del marco consolidado propuesto para la IR.

Con base en lo anterior, el objetivo de este artículo fue realizar una revisión sistemática de los marcos o modelos conceptuales que incorporen, de manera explícita, aspectos de equidad en salud en la investigación de la implementación (IR), con el fin de conocer y describir la forma en la que se incluyen los aspectos de equidad en las diferentes propuestas y, a partir de allí, evaluar la necesidad de proponer un marco consolidado de IR en el que se incluyan los aspectos de equidad en salud.

## MÉTODOS

Se llevó a cabo una revisión sistemática de la literatura, que incluyó una estrategia de búsqueda sistemática y exhaustiva en las bases de datos Medline (Pubmed), Embase y Lilacs, entre 1965 y octubre de 2016, y Scopus entre 1998 y octubre de 2016 (ver Anexo 1). De igual manera, se utilizó una estrategia en bola de nieve a partir de las referencias y se incluyó literatura gris adicional. Se incluyó, además, una revisión de sitios web de entidades que hacen IR como el de la Alianza para la Investigación en Políticas y Sistemas de Salud, la Organización Mundial de la Salud (OMS), la OPS, *The National Implementation Research Network, The Training Institute on Dissemination and Implementation Research, The Implementation Research Institute, Society for Implementation Research Collaboration y United States Agency for International Development* (USAID). Todos los títulos relacionados fueron incluidos luego de eliminar los duplicados. No se realizó exclusión por el idioma. Tres revisores independientes calificaron los artículos no relevantes y categorizaron los artículos en los cuales se propusieran o presentaran marcos o modelos conceptuales en los que relacionaran elementos de equidad en salud e IR.

No se evaluó la calidad de los artículos, dado que la pretensión fue recoger los diferentes componentes metodológicos que han incorporado el tema de equidad en salud en IR bajo un marco conceptual y, por ello, no son estudios de investigación que puedan ser evaluables.

## RESULTADOS

**FIGURA 1.**
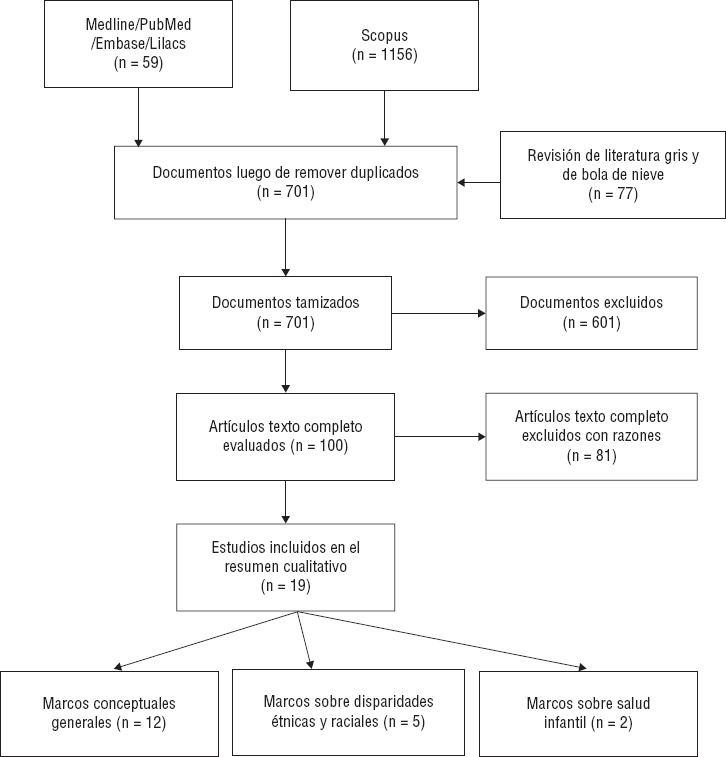
Detalles de la búsqueda sistemática de literatura.

Los resultados de la búsqueda sistemática arrojaron finalmente 701 artículos (figura 1) luego de eliminar duplicados. Se incluyeron 100 artículos para revisión en texto completo. Por último, 19 artículos trataron sobre marcos conceptuales con elementos de equidad en IR. De estos, 12 fueron marcos generales, cinco fueron marcos para disparidades étnicas o raciales y dos se relacionaron con salud infantil (ver Anexo 2).

**FIGURA 2.**
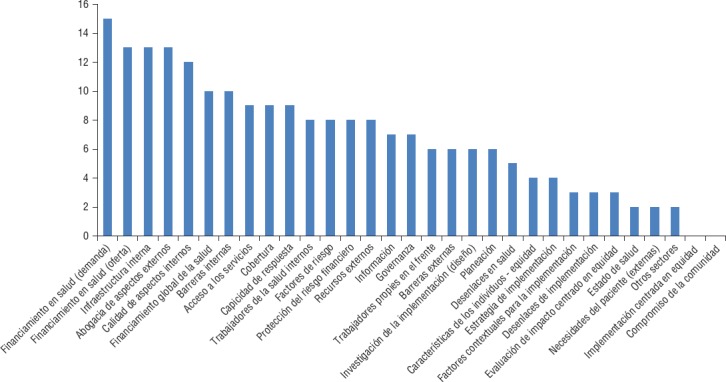
Categorías relacionadas con mayor frecuencia en los marcos conceptuales.

En la figura 2 se aprecian como categorías más frecuentes en los marcos conceptuales encontrados: financiación, infraestructura y abogacía, calidad, barreras internas y cobertura. Las categorías menos frecuentes fueron: otros sectores, las necesidades externas de los pacientes, el estado de salud y la evaluación del impacto sobre la equidad. No hubo mención alguna sobre el compromiso de la comunidad y del proceso de implementación.

En el cuadro 1 se presenta el resumen de los aspectos generales de los marcos conceptuales y su relación con la equidad.

A continuación se describen algunos de los modelos más relevantes:

El modelo de Marco nacional de monitoreo, evaluación y análisis del sector salud (MNMEASS) (8) hace énfasis en el monitoreo de las desigualdades en salud y en las recomendaciones para promover la equidad y se centra en las enfermedades de los pobres, en la implementación de servicios donde viven los pobres y en la eliminación de barreras financieras mediante la utilización de metas y monitorización con lentes de equidad (ver Anexo 3).

En el Marco para la evaluación de impacto (IAF por sus siglas del inglés *Impact Assessment Framework*) (9) se incluye la equidad como una de las cinco capas de análisis que se debe considerar en la evaluación de impacto y se centra en quienes se benefician de la implementación; es decir, si la prueba o intervención es igual de exacta para todos los pacientes, qué costos enfrentan los pacientes, qué tan aceptable es la prueba o intervención para los pacientes. Sin embargo, al momento de la evaluación no mencionan todas las potenciales variables que podrían desencadenar desigualdades e inequidades en salud en la población, solo se centran en el acceso, valores y costos potenciales para los pacientes.

En la propuesta explícita del Marco para el fortalecimiento de los sistemas de salud (FHSS por sus siglas del inglés *Framework for Health System Strengthening*) (10), se incluyen de manera separada el mejoramiento de la salud, el mejoramiento de la equidad, y adicionalmente, la protección del riesgo financiero y social. Una limitación de esta última es, por ejemplo, el aumento de manera indirecta de la inequidad en salud, en los casos que dicha protección sea diferencial, a favor de quienes tienen mejor capacidad de pago.

En el marco conceptual de PARiHS (del inglés *Promoting Action on Research* *Implementation in Health Services*) (11), se mapean de manera gráfica el papel de la evidencia y el contexto (fuerte o débil), en la implementación (ver Anexo 3). A pesar de ser una propuesta bastante práctica, no se menciona el uso de evidencia o factores contextuales relacionados con la afectación de las inequidades en salud. De hecho, hay escenarios en el marco de equidad en salud en los cuales la evidencia puede ser débil por la falta de estudios apropiados para favorecer la implementación de una intervención, pero el contexto exige que se implemente una intervención.

La estrategia CHNRI (del inglés *Child* *Health and Nutrition Research Initiative*) (12) está enfocada en definir las prioridades de investigación en salud, en donde se utilizan como elementos para calificar la priorización: el efecto sobre la equidad. Se discute el peso relativo asignado a cada una de las categorías para priorizar, pues se asume que este es igual a todas las categorías, incluida la de equidad. Sin embargo, con frecuencia se prefieren las intervenciones que son más fáciles de entregar, o en las que se tiene mayor capacidad de respuesta, sin importar el efecto sobre la equidad. Si se prefiere impactar de manera positiva sobre la equidad, esta variable debería tener un mayor peso al momento de priorizar o incluir consideraciones de equidad al instante de definir la población (población en desventaja), o de evaluar el impacto sobre la carga de enfermedad (en esta población en desventaja).

El Modelo de disparidades étnicas y raciales *(Conceptual Model on Racial and Ethnic Disparities in Health Care)* (13) es teórico y sirve más para identificar actores y acciones potenciales en la generación o disminución de las potenciales disparidades. En él se involucran instituciones gubernamentales y no gubernamentales, los pacientes, las personas alrededor de los pacientes, la comunidad, las normas sociales y el contexto alrededor del paciente y alrededor del sistema de salud (ver Anexo 3).

El modelo de Consideraciones de equidad en la implementación a gran escala de la fortificación de condimentos y especias (ISECLSFCS por sus siglas del inglés *Implementation strategies and equity* *considerations for large-scale fortification of* *condiments and seasonings*) (14) aporta varios puntos específicos para la implementación considerando la equidad, por ejemplo, las barreras en la implementación de acciones multisectoriales, es clave en la afectación de los determinantes sociales, para todas las intervenciones, servicios y programas en la que esto se considere (14).

En la extensa propuesta del Manual del conocimiento a la acción: promoviendo la acción en aspectos de equidad (*Promoting action on equity issues: a knowledge-to-action handbook*) (3), se proponen tres fases: la primera para entender y delimitar el problema (lo que se sabe, qué evidencia se necesita, quién determina la evidencia para ser incluida y las barreras para usarla); la segunda propone abrir un espacio en la agenda de los tomadores de decisiones para presentarles la evidencia; y la tercera para informar la respuesta, en la que se vuelve a proponer la preparación de la respuesta centrada en los tomadores de decisiones, aunque se incluyen consideraciones locales, de comunidad, de recursos y de tiempo para facilitar el proceso de convencimiento ante los tomadores de decisiones. Como se aprecia, se hace un gran énfasis en los tomadores de decisiones aunque olvidan incluir a otros actores que podrían ejercer función como barreras o facilitadores para la implementación, a nivel de la comunidad y de los implementadores mismos.

El marco de la propuesta de *The Australasian Collaboration of equity-focused health impact assessment* (ACEFHIA) (15) se centra en la preparación y toma de decisiones para dar recomendaciones que afecten la equidad en salud de tal manera que, de los seis pasos sugeridos, los cinco primeros llegan a la recomendación, pero no son explícitas las “Recomendaciones para la implementación”, lo que es un problema para este modelo, que pasa sin más explicaciones al monitoreo y la evaluación (ver Anexo 3). 

***Fuente:*** elaborado por los autores.

La herramienta de evaluación rápida del impacto en las desigualdades en salud *Health Inequalities Impact Assessment Rapid Appraissal Tool* (HIIARAT) (16) provee elementos para tomar la decisión de implementar intervenciones, posteriores a la evaluación rápida de impacto y de la afectación positiva de las desigualdades en salud (ver Anexo 3), pero no da elementos concretos que sugieran como efectuar la implementación para mantener el impacto esperado.

El marco de la auditoria de la salud *(Equity audit of health services)* (17, 18) otorga orientación para planificar las acciones de los tomadores de los decisiones y, aunque tiene un fuerte foco sobre la equidad, no proporciona elementos que orienten la implementación hacia una mayor disminución de las inequidades en salud.

En el Anexo 3 se incluye un mayor detalle de estos y otros marcos conceptuales.

## DISCUSIÓN

Este artículo identificó, luego de una búsqueda y síntesis sistemática, documentos que enlazaban el concepto de investigación de la implementación con equidad en salud bajo el enfoque de un marco o modelo conceptual. Se encontraron varios marcos o propuestas conceptuales que incluían el tema de equidad en salud, quizá relacionado con el hecho de la necesidad de resolver la brecha entre el conocimiento y la acción (o el servicio), como lo menciona Bowen (3).

Sin embargo, fueron pocos los marcos o modelos conceptuales que especificaron con claridad cómo llevar la equidad en salud al plano de la implementación. Unos lo hacen a través del monitoreo de las desigualdades en salud y sus recomendaciones (MNMEASS); otros generan recomendaciones como parte de los resultados obtenidos a partir de la IAF y el FHSS; por último, otros toman acciones en el proceso de implementación, sobre los factores que afectan la implementación enfocada en la equidad en salud: ISECLSFCS, el Modelo de disparidades étnicas y raciales y el Manual del conocimiento a la acción.

Además, para disminuir las inequidades en salud, se han plasmado objetivos, metas, estrategias, programas e intervenciones en diferentes países o entidades internacionales. Un ejemplo de esto son las Metas de Desarrollo Sostenible; en ellas, la igualdad de género y la reducción de desigualdades entre las naciones hacen parte de las 17 metas proyectadas para el 2030 (19). Los marcos y propuestas identifican de manera general puntos comunes que se repiten en mayor o menor frecuencia a través de las diferentes categorías de los artículos encontrados. Aspectos como la financiación de la salud en las propuestas de implementación (sobre todo de la demanda), el papel de los profesionales de la salud, (sobre todo los responsables de la implementación), la gobernanza, el acceso y la abogacía, son categorías frecuentes en esta revisión.

El principal punto común de la implementación en estos modelos es la acción en la provisión del servicio, política o programa. Desde la perspectiva de este estudio, así como se proyecta disminuir la brecha entre la efectividad esperada de la intervención y la encontrada en la población (3, 20), se esperaría que lo mismo ocurriera con el efecto deseado sobre la disminución esperada de las inequidades en salud en cada uno de los modelos encontrados llevados a la práctica real.

**CUADRO 1.** Resumen de marcos conceptuales incluidos sobre investigación de implementación y equidad en salud

Autor	Categorías y detalles	Aspecto relacionado con la equidad
OPS/OMS (1)	Marco nacional para el seguimiento, la evaluación y el análisis del sector de la salud. Propone reconocer que el sector salud es parte del problema, priorizar las enfermedades de los pobres, implementar o mejorar los servicios donde viven los pobres mediante la utilización de canales de distribución adecuados, reducción de las barreras financieras para el cuidado de la salud y establecimiento de metas y su monitoreo con perspectiva de equidad.	Ingreso, educación, género, etnia
Mann G, et al, 2010 (2)	Marco de evaluación del impacto, con cinco niveles de análisis: eficacia, equidad, sistema de salud, análisis de políticas y escalamiento.	General
Mann G, et al, 2011 (3)	Marco para el fortalecimiento del sistema de salud mediante la utilización de la perspectiva de la investigación en economía de la salud. Se basa en el marco previo presentado por Mann, pero enfatizan en el uso de herramientas de economía de la salud. En el caso de la equidad, proponen evaluar la ayuda financiera para facilitar el acceso y la protección financiera.	General
Kitson AL, et al, 2008 (4)	Marco para la promoción de la acción en la Investigación de Implementación en los servicios de salud (PARiHS): fase de preprueba diagnóstica, puntuaciones resumidas de la evidencia (E) y el contexto (C), resumen narrativo, información sobre los prototipos de enfoques de facilitación, el proceso de facilitación, la evaluación posterior a la prueba, resume las puntuaciones de resumen para E + C, resumen narrativo, evaluación del enfoque de facilitación.	General
Rudan I, 2016 (5)	La Iniciativa de Investigación de Salud Infantil y Nutrición (CHNRI), con un marco para obtener ideas de investigación, un enfoque sistemático para discriminar dependiendo del contexto formado por la población de interés, la carga la enfermedad de interés, los límites geográficos, la escala de tiempo y el estilo preferido de Invertir con respecto al riesgo. Los cinco criterios para priorizar son: capacidad de respuesta, efectividad, capacidad de entrega, máximo potencial de reducción de la carga de morbilidad y efecto sobre la equidad.	Salud infantil
Chin MH, et al, 2007 (6)	Es un modelo explicativo y teórico de disparidades étnicas y raciales, enfocado en actores y acciones para generar o disminuir disparidades.	Etnia
Bailie RS, et al, 2007 (7)	Modelo de Implementación de la Herramienta de Promoción de la Salud Indígena que fue utilizado por Percival en 2016 (8). Tiene seis pasos, cuatro de ellos cíclicos: evaluación de calidad, interpretación participativa, retroalimentación y planificación para la acción y la implementación. Centrado en la población indígena.	Etnia
Zamora G, et al, 2016 (9)	Marco conceptual de consideraciones de equidad para la implementación en una estrategia de fortalecimiento a gran escala de condimentos, con base en fortalecer las capacidades del sector de salud pública, mejorar el desempeño de los organismos de ejecución, fortalecer las capacidades y el desempeño de los trabajadores de primera línea, empoderar comunidades e individuos, apoyar a múltiples actores involucrados en la mejora de la salud.	General
Bowen, S. et al, 2011 (10)	Conjunto de herramientas de transferencia de conocimientos (11): se desarrolló a partir de la propuesta de promover acciones sobre aspectos de equidad en un Manual de conocimiento para la acción. Las categorías para la implementación son: implementación de reportes (categoría 2.4), cambiar la práctica (categoría 2.5), mantener el apoyo (fase 6) y tener clara la audiencia (categoría 3).	General
NHS-Scotland, 2005 (12)	La herramienta de diez pasos para evaluar el impacto sobre la igualdad y la diversidad, que permite monitorear el impacto sobre la igualdad y diversidad, de las políticas y actividades del sistema de salud.	General
Mahoney M, et al, 2004	Marco de evaluación del impacto centrado en la equidad en salud. Propone los siguientes pasos: tamizaje, alcance, identificación del impacto, evaluación del impacto, recomendaciones, y monitoreo y evaluación.	General
Scott-Samuel A, 2001 (13)	Las Guías Merseyside para la Evaluación del Impacto en Salud. Marco de procedimientos y métodos para ser usados durante la implementación. El énfasis en la equidad ocurre cuando la recomendación dada es considerada (equidad social y aceptabilidad) y cuando declaran sus métodos al público, en el cual deben considerarse los valores. Se menciona que: “el enfoque centrado en la equidad es el uso de métodos participativos” en todas las etapas de la evaluación para esta propuesta.	General
Bro Taf Health Authority, 1999 (14)	Herramienta de evaluación rápida en el impacto sobre las desigualdades en salud. Tiene cinco secciones: lluvia de ideas, evidencia, oportunidades, evaluación del impacto, y detalles de la evaluación y del monitoreo.	General
Johnstone F, et al, 1996. (15)	Auditoría de la equidad de los servicios de salud. Es un marco de planificación y priorización del Sistema Nacional de Salud en el Reino Unido. Utiliza nueve preguntas para orientar los planes de acción futuros en torno a las desigualdades, la priorización, los programas actuales, las metas, las acciones, los recursos y los impactos observados sobre las desigualdades.	General
Global Equity Gauge Alliance, 2003 (16)	Marco para la acción con tres pilares: evaluación y seguimiento, abogacía y empoderamiento de la comunidad.	General
Kilbourne AM, et al, 2006 (17)	Marco conceptual para el avance de la investigación sobre disparidades en la salud dentro del sistema de salud. Consta de tres fases: detectar las disparidades, comprenderlas y reducirlas. En esta última fase se propone desarrollar la intervención, evaluar el desempeño de la intervención, implementación y transferencia utilizando una estrategia de tres pasos: programa de mejora de la calidad, evaluar el esfuerzo de implementación y refinamiento de la intervención para una amplia difusión, y colaboración entre investigadores de la comunidad, para facilitar el proceso de implementación.	General
Chin MH, et al, 2014 (18)	Investigación de disparidades para el cambio. Incluye: 1) la abogacía de la equidad, para los estudiantes y el personal, 2) la construcción de habilidades para buscar soluciones a nivel de sistema a las barreras para los pacientes, 3) incorporar la equidad en la enseñanza sobre la mejora de la calidad y el aprendizaje basado en la práctica, 4) determinar cómo encaja la equidad y la responsabilidad social en nuestros planes, como parte de la cultura organizacional para la equidad, y 5) participación de la comunidad.	Etnia
Peters DH, et al, 2013 (19)	Marco consolidado para la investigación de la implementación que considera cinco elementos: características de la intervención, el entorno externo, el entorno interno, las características de los individuos involucrados, y el proceso de implementación. Aunque se menciona la equidad como un tema a mejorar con la investigación de la implementación, no hay una manera clara de incluirlo en el marco.	General
Rollins N, et al, 2014 (20)	Se utilizó la Iniciativa de Investigación sobre Nutrición de la Salud Infantil y se añadieron preguntas para facilitar la priorización de las preguntas de investigación de la implementación: (1) que se pueda responder con la investigación, (2) con probabilidad de reducir las infecciones pediátricas por VIH, (3) abordaje las principales barreras del escalamiento, (4) innovación y originalidad, (5) mejoría de la equidad entre las poblaciones desatendidas y (6) probablemente útil para los formuladores de políticas. También incluye población vulnerable, y una de las preguntas está relacionada con la equidad. Es un marco para la priorización.	Salud infantil

***Fuente:*** autores de varias referencias.

En ese sentido, la Asociación Americana de Salud Pública ha propuesto una guía para facilitar la implementación de Salud en todas las Políticas (21), que armoniza la necesidad de generar acciones desde otros sectores para mejorar la salud de la población. Para la OMS y la OPS; también es fundamental lograr el acceso y la cobertura en salud universal, para llegar al máximo estado de salud posible (22). En este contexto no hay lugar para las brechas que existen hoy en día en los resultados en salud entre los países, y peor aún, al interior de los paí- ses (19, 23-25).

Una revisión narrativa de literatura previa describió marcos conceptuales, modelos y teorías relacionados con la IR, pero no con las consideraciones específicas de equidad en salud (26). Por otra parte, la Organización Mundial de la Salud propone el modelo Innov8 que hace énfasis en la población excluida de los beneficios de los programas de salud y los mecanismos que generan las inequidades en salud para ser abordados durante la implementación (27). Aunque es una propuesta muy bien desarrollada, no incluye un marco evaluativo de IR. Para disminuir las brechas en salud, y con ellas las inequidades en salud, se hace necesario contar con un marco consolidado de IR en el que se incluyan los aspectos de equidad en salud. Dicho marco facilitaría mejorar los procesos de implementación de intervenciones, servicios o programas de salud, tomando en cuenta los factores contextuales que afectan o podrían afectar los propios procesos de implementación (28). La Comisión sobre los Determinantes Sociales de la Salud (CSDH) de la OMS destaca el importante papel que desempeña el monitoreo en la mejora de la equidad en salud mediante la recomendación de “medir y entender el problema y evaluar el impacto de la acción”. Para aplicar las recomendaciones de la resolución de la OMS en todas las esferas de acción, incluida la supervisión, los países requieren evidencia sobre los programas de políticas que abordan los determinantes sociales de la salud y mejoran la equidad sanitaria.

Para integrar la igualdad en salud en los programas de salud, la investigación de la implementación se centraría en marcos conceptuales que consideren las interrelaciones y la relación causa-efecto entre indicadores y dominios sociales, teniendo en cuenta de manera específica los indicadores de salud y las desigualdades. Esto permitirá a los responsables de la formulación de políticas crear programas de salud que apunten a políticas y acciones intersectoriales.

El objetivo de equidad en salud debe aplicarse a las diferentes etapas de implementación de las actividades de investigación y promover la implementación de los programas de salud. Dado que es esencial contar con una imagen fiable y clara de cómo se distribuyen las oportunidades de salud y salud en una población determinada y qué factores contribuyen o reducen las oportunidades para ser saludables, los resultados de la investigación también deben ser analizados y usados con una perspectiva de equidad en la salud para reducir la inequidad en la salud entre las poblaciones. De esta manera, se apoyan los objetivos 3 y 10 de desarrollo sostenible: garantizar vidas saludables y promover el bienestar de todos en todas las edades, y reducir las desigualdades dentro y entre los paí- ses, respectivamente.

Por último, todo lo anterior conlleva a un cuestionamiento ético cuando las intervenciones o programas se implementan sin considerar su potencial impacto sobre la equidad en salud en lo que respecta a beneficios, cobertura y riesgos potencialmente diferenciales en la población (28): esto amplía las brechas en salud y, con ellas, de las inequidades en salud.

Por tratarse de un estudio basado en fuentes secundarias, tiene los potenciales sesgos relacionados con la no publicación de artículos que, si bien relevantes, no se incluyeron en las bases de datos revisadas. Aunque la intención fue solo conocer las propuestas de marcos conceptuales, se incluyeron estudios con diferente nivel de calidad metodológica que no fue posible evaluar por no tratarse de propuestas de investigación formales.

## CONCLUSIÓN

Aunque a nivel mundial se promueve la disminución de las desigualdades y, en particular, las desigualdades en salud, este tema aparece muy tímidamente en los marcos conceptuales publicados sobre la IR. Durante la búsqueda se hallaron pocos documentos sobre marcos conceptuales y una baja frecuencia en lo referente a la evaluación del impacto sobre la equidad. A partir de esta revisión, se sugiere replantear un marco de equidad en salud en el escenario de la IR, considerando los aspectos generales que abarcan la investigación de la implementación y que se articulan contextualmente con los fenómenos locales que afectan de mayor o menor manera el impacto final de las intervenciones o programas sobre la equidad en salud. Un marco conceptual que articule estos aspectos generales y específicos ayudará en la implementación de las intervenciones, políticas y programas en sus respectivos contextos. Esto ayudará a disminuir la brecha entre la efectividad esperada y lograda sobre la equidad en salud. El enfoque de equidad dentro de un marco conceptual es fundamental en la investigación de la implementación para dirimir estos aspectos.

### Agradecimientos

Los autores desean agradecer a la diseñadora gráfica editorial Luisa Fernanda Florián, quien diagramó las figuras del Anexo 3.

### Declaración

Las opiniones expresadas en este manuscrito son responsabilidad del autor y no reflejan necesariamente los criterios ni la política de la RPSP/PA- JPH y/o de la OPS
